# A Review on the Respiratory System Toxicity of Carbon Nanoparticles

**DOI:** 10.3390/ijerph13030325

**Published:** 2016-03-15

**Authors:** Maricica Pacurari, Kristine Lowe, Paul B. Tchounwou, Ramzi Kafoury

**Affiliations:** 1 Biology Department, College of Science, Engineering, and Technology, Jackson State University, Jackson, MS 39217, USA; paul.b.tchounwou@jsums.edu (P.B.T.); ramzi.kafoury@jsums.edu (R.K.); 2 Research Center in Minority Institutions-Center for Environmental Health, College of Science, Engineering, and Technology, Jackson State University, Jackson, MS 39217, USA; 3 Biology Department, The University of Texas Rio Grande Valley, Edinburg, TX 78539, USA; Kristine.Lowe@utrgv.edu

**Keywords:** CNF, CNT, respiratory pathology, toxicological pathways, exposure levels

## Abstract

The respiratory system represents the main gateway for nanoparticles’ entry into the human body. Although there is a myriad of engineered nanoparticles, carbon nanoparticles/nanotubes (CNPs/CNTs) have received much attention mainly due to their light weight, very high surface area, durability, and their diverse applications. Since their discovery and manufacture over two decades ago, much has been learned about nanoparticles’ interactions with diverse biological system models. In particular, the respiratory system has been of great interest because various natural and man-made fibrous particles are known to be responsible for chronic and debilitating lung diseases. In this review, we present up-to-date the literature regarding the effects of CNTs or carbon nanofibers (CNFs) on the human respiratory system with respect to respiratory toxicity pathways and associated pathologies. This article is intended to emphasize the potentially dangerous effects to the human respiratory system if inadequate measures are used in the manufacture, handling, and preparation and applications of CNP or CNP-based products.

## 1. Introduction

Carbon nanomaterials, mainly represented by carbon nanoparticles or carbon nanotubes (CNPs/CNTs), and carbon nanofibers (CNFs), have captured the interest of the scientific community for more than two decades since the discovery of the first nanometer-sized carbon nanotubes [1]. Carbon nanomaterials have unprecedented potential for a large variety of industrial and biomedical applications. In industry, carbon-based nanomaterials have potential uses in many applications ranging from aerospace, construction, automobiles, electronics, and others. In the medical field, carbon nanomaterials are being considered as contrast agents, chemo-carriers, biological platforms, and for many other applications [2]. Currently, financial investment in nanotechnology is substantial, suggesting that an abundance of nano-based products will reach the market in the next few years [3]. While CNTs have been the focus of intense research, another type of carbon-based nanomaterial, CNF, is becoming increasingly prevalent as a nanomaterial of choice for nanoproducts/nanoscaffolds. CNFs seem to be a very suitable nanomaterial for biomaterial production. However, their effects on biological processes are mostly unknown to date. Thus, this review aims to present the current information on CNPs/CNTs and CNFs effects on the respiratory system.

## 2. Are CNT and CNF Structurally Different?

Carbon allotropes are present in a variety of carbon nanostructures including graphene, amorphous carbon, CNTs, and CNFs. Like CNTs, CNFs are increasingly used in many industrial applications, particularly, as polymer nanocomposites to provide strength, stiffness, heat resistance, and durability [4]. CNTs and CNFs belong to the same family, yet both these carbon nanostructures have distinct features. The physical and chemical properties of CNTs have been comprehensively presented in numerous reviews [5,6] and will not be discussed in the present review. Single-walled (SW)-CNTs are more distinct from CNFs, as opposed to multi-walled (MW)-CNTs. SWCNTs are a single sheet of graphene and, therefore, possess less rigidity and aggregate in bundles, properties that set them apart from MWCNTs and CNFs. MWCNTs are multiple sheets of graphene arranged concentrically with diameters between 10 and 150 nm [7]. Structurally, CNFs are layers of graphene arranged in an axial arrangement that form these carbon nanostructure fibers. CNFs have diameters ranging from 70 to 200 nm, and lengths of 10 to 100 μm [8]. CNFs may pose different health hazard implications compared to CNTs due to the differences in structural aspects between CNTs and CNFs; moreover, these nanostructures are less investigated than CNTs. It is envisioned that CNT and CNF end-products will dominate many aspects of industry due to their attractive intrinsic properties; thus, increased human exposure either at the synthesis or end-product is highly possible. Therefore, understanding the toxicological and pathological effects related to CNTs and CNFs is imperative to avoid potential respiratory effects such as those observed, for example, with asbestos exposure [5,9,10].

## 3. CNTs and CNFs as Bio-Scaffold and Bio-Sensor Materials

CNTs and different CNFs nanostructures are being investigated as biomaterial hybrid scaffolds. The sustainability of CNFs as scaffold materials stems from their fibrous nature, which mimics the fibrillar structure of extracellular matrix proteins. This nano-fibrillar structure provides a structural mesh for cell adherence and interactions making CNFs potential nanomaterials for orthopedic applications and more recently for peripheral nerve regeneration [11,12]. For example, a type of CNF with a hat-stacked nanostructure (H-CNF) were implanted subcutaneously, and after four weeks these H-CNF clusters were surrounded by a thin layer of connective tissue suggesting no tissue toxicity but indeed biocompatibility with surrounding tissue [12]. However, slight inflammation and the presence of phagocytizing macrophages were noticed at the H-CNF site. These processes were however considered as part of the wound healing process rather than a toxicological response. Carbon nanofiber-based thin films and mats have been tested for directed growth of Schwann cells [11]. Schwann cells proliferated when grown on CNF- and carbon-films without apoptosis or reactive oxygen species (ROS) formation. These results suggest that the fibrous nature of CNF provides suitable physical guidance for peripheral nerve cell growth, and may open-up new ways for neural tissue engineering, particularly peripheral nerve regeneration [11].

Cell adherence to CNFs patterned on polymer substrates was evaluated using osteoblast cells. Khang *et al.* [13] maintained cultures of osteoblasts for 21 days on matrices patterned with CNFs and found osteoblast cells to be fully functional with mineral deposition along the CNF patterns. Price *et al.* [14] used two types of carbon fiber compacts, conventional carbon and nanometer carbon fibers, to determine the adhesion of osteoblast cells. Osteoblasts cultured on carbon nanofiber scaffolds showed selective adherence to nanometer-dimension rough surfaces. These results suggest that CN-based scaffolds, perhaps due to their nanosize, are the preferred nanomaterials for use in directed bone growth applications, or for successful orthopedic and dental implants.

MWCNTs have been tested for muscle tissue engineering. Ostrovidov *et al.* [15] investigated a hybrid of gelatin and multi-walled fibers for myotube formation and functionality. MWCNTs enhanced the formation of fully functional myotubes with mechano-transductive properties, suggesting that MWCNTs may represent a novel scaffold material for skeletal tissue formation.

SWCNTs and MWCNTs have been tested as biosensors for the detection of proteins, nucleic acids, enzymes, and other biomolecules [16,17]. Carbon nanofibers, just recently, have been used for biosensors. For example, vertically aligned carbon nanofibers (VACNFs) seem to be a suitable material for the fabrication of biosensors for the detection of cardiac troponin [18] or C-reactive protein (CRP) [19]. Gupta *et al.* [19] tested a CNF-based biosensor platform for the detection of CRP and demonstrated that a CNF-sensor could potentially be developed to detect clinically relevant CRP level with high specificity. Moreover, the applicability of CNF-based biosensors has been extended to evaluate drug metabolism and inhibition. Xue *et al.* [20] reported the development of a CNF-modified film electrode in which the drug metabolizing enzyme, CYP3A4, was physically adsorbed producing a CYP3A4/CNF-modified film electrode. In comparison with CNT or carbon black (CB), the CNF-modified film electrode was the most efficient substrate for the physical adsorption of CYP3A4 onto film. The application of CNF-based film electrodes has the potential to revolutionize the time and type of drug metabolism screening as well as screening for CYP3A4 inhibitors. CNF applications have also been extended to phyto-pharmaceutical testing with potential applications in anticancer phytochemical discovery. Shao *et al.* [21] developed MWCNT-based nanofibers to which green tea polyphenols were adsorbed to test green tea antioxidant cancer therapy. The viability of an array of cell types (e.g., osteoblast, A549, and HepG2) on GTP-bound nanofibers were tested. Of all tested cells, HepG2 cells were more sensitive to GTP-bound nanofibers compared to osteoblast or A549 cells, suggesting the appropriateness of carbon-nanofiber platforms for antioxidant-linked cancer therapy. The applicability of CNF-based biosensors has been tested for the detection of catechol, a phenolic compound found in pesticides, and other pharmaceuticals [22]. The detection of catechol in solution was possible with the use of the laccase enzyme immobilized on electrospun CNF (ECNF) films. The detection limit of catechol in the solution was 0.63 μM within a 2 s response time, an indicator that such platforms could revolutionize testing for environmental/food chemistry safety.

Another recent application of carbon nanoparticles was the use of CNTs for microRNA (miRNAs) detection. Li *et al.* [23] designed MWCNT-based biosensors to which synthetic DNA probes complementary to miRNAs were immobilized. The hybridization of specific microRNAs was measured using guanine oxidation signals when a specific microRNA bound to its complementary DNA. Guanine oxidation was quantified using differential pulse voltammetry. Detection of microRNA expression using MWCNT-biosensors appears to be simple and cost-effective, given that microRNA are emerging important regulators of gene expression in plants, animals, and humans. Therefore, determining microRNA expression levels will provide information that will help to understand how microRNAs are associated with biological processes and human disease. To validate this method, Li *et al.* [24] tested microRNA-24 hybridization to the MWCNT-biosensor, which showed a good sensitivity, selectivity, and reproducibility. However, hybridization time seemed to play an important role in the detection of microRNA-24, suggesting that microRNA are susceptible to degradation. Despite this, the authors were able to obtain an acceptable recovery concentration of microRNA-24.

MWCNTs applications to biological systems, such as skeletal muscle fiber formation on gelatin nanofibers-MWCNT hybrid scaffolds, have been also investigated. Ostrovidov *et al.* [15] examined two concentrations of MWCNT, 0.5 and 5 mg/mL, combined with gelatin (20%) as a scaffold support for myofiber formation. Addition of MWCNT significantly increased the mechanical strength of gelatin nanofibers according to Young’s modulus measurements, and provided the necessary frame for C2C12 myoblast cell alignment, an indication for supporting myotube formation. C2C12 cell viability was not affected when the cells were cultured for two days, and myotube length increased with increasing MWCNT concentrations on gelatin nanofibers. Moreover, the gelatin-MWCNT nanofiber-guided myotubes exhibited contractibility under electrical stimulation that increased with increasing MWCNTs concentrations. These results suggest that gelatin-MWCNT hybrid scaffolds may be useful for skeletal muscle formation which could be applicable for muscle regeneration in skeletal muscle diseases.

CNTs/CNFs have the potential to function as biosensors for respiratory system pathological diagnosis, such as their use for the detection of lung cancer. Choudary *et al.* [25] showed that lung cancer antigens, MAGE A2 and MAGE A11, covalently attached to SWCNT-chitosan composites detected anti-MAGE A2 and anti-MAGE A11, respectively. A more sophisticated application of CNT-based biosensors for diagnosis and detection was developed by Tian *et al.* [26]. Using CNTs and hairpin sequences against a specific microRNA, Tian and coworkers showed femtomolar detection of the lung cancer biomarker, let-7 microRNA.

These studies indicate the possible applicability of using CNTs/CNFs as bioscaffolds in regenerative medicine and biosensors in clinical assays. Although CNTs induce toxicological responses in the respiratory system in cellular and animal models, CNTs in combination with biopolymers seem to have different interactions, particularly, with osteogenic or neuronal cells. Moreover, since most of these studies were short term, it is prudent to suggest the need for long-term studies for the applicability of CNTs/CNFs as bioscaffolds.

## 4. CNT Toxicological Pathways

### 4.1. Oxidative Stress

**Figure 1:**
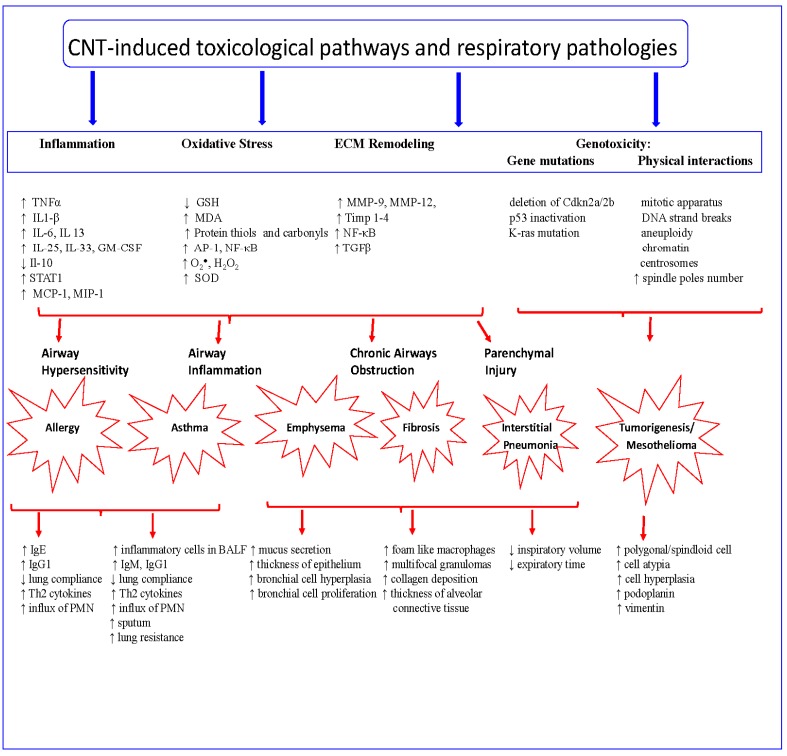
CNT-induced toxicological pathways and the associated respiratory pathologies. CNT induce upper and lower respiratory system pathologies in diverse experimental models. The upper part of the figure indicates the toxicological pathways induced by CNT either *in vitro* or *in vivo* studies. The shown respiratory pathologies and associated physiological changes in the tissues or biological fluid are those observed in numerous animal studies. ↑, increased expression or increased number; ↓, decreased expression; the long arrow indicated the induction of respective toxicological pathways, pathologies, and associated markers. This figure was prepared from the extensive review of the studies cited in this manuscript and presented in the Reference section.

Oxidative stress is the most accepted mechanism for CNTs, asbestos, and other nanoparticles’ toxicity [5,27,28,29,30]. It is well established that ROS can react with cellular macromolecules and disrupt intracellular homeostasis. For example, numerous studies have shown that the presence of transition metals (e.g., Fe, Ni, Cu) in CNTs induces the formation of molecular oxygen-dependent superoxide anion radicals (O_2_∙^−^), hydrogen peroxide (H_2_O_2_) and hydroxyl radicals (∙HO), all of which have high redox potentials and reactivities [29,30] (Figure 1). However, studies have also shown that even purified MWCNTs induce cyto- and genotoxic responses in BEAS-2B bronchio-epithelial cells [31,32]. Specific molecular signaling factors associated with oxidative stress, such as activator protein-1 (AP-1), nuclear factor κB (NF-κB), and MAP kinases ERK1/2 and p38, are activated by CNTs/CNFs and lead to antioxidant defense mechanism depletion or downregulation [32]. In mesothelioma cells, MWCNTs induced the formation of superoxide and DNA damage [33]. *In vivo* studies by Khaliullin *et al.* [34] and Shvedova *et al.* [35] showed that MWCNTs given by aspiration or SWCNTs either administered by pharyngeal aspiration or inhalation, decreased the level of glutathione, increased the level of protein thiols, and increased the level of malondialdehyde (MDA). Although CNFs are similar to MWCNTs, CNFs’ specific physicochemical characteristics, particularly having a low level of catalytic trace metals, may or may not induce ROS. This fact still requires further toxicological experimental investigation.

### 4.2. Extracellular Matrix Remodeling/Tissue Remodeling

Tissue remodeling of the respiratory system is a key pathophysiological characteristic of lung diseases including asthma, emphysema, and fibrosis. Mechanistically, airway remodeling is associated with biological processes such as cellular structural changes, cell proliferation, cell hyperplasia, cell hypertrophy, subepithelial fibrosis, inflammation and vascular changes [36]. The role of CNTs in lung remodeling has been shown by numerous studies which have demonstrated how they induce respiratory system remodeling via an array of cellular and molecular factors that favor the tissue remodeling processes. For example, CNTs alone or in allergen-sensitized animal models foster airway and lung remodeling via cytokines TNFα, IL1-β, MCP-1, Il-13, and recruitment of blood inflammatory cells [35,37,38,39,40,41]. Extracellular remodeling induced by CNTs is mediated primarily via TGFβ, TNFα, and osteopontin (OPN) signaling pathways [34,42]. Although TGFβ is a key cytokine that regulates extracellular matrix remodeling via STAT1, Thompson *et al.* [40] showed a role of STAT1 in MWCNT-induced airways remodeling. However in STAT1-depleted and OVA-sensitized mice or house-dust mites-sensitized mice, MWCNTs increased collagen, TGFβ, Il-13, eotaxin, and mucus production. These results suggest that other cytokines compensate for the TGFβ signaling pathway [40,43]. TGFβ1 is a key molecule in the development and progression of pulmonary fibrosis. It promotes the transcription of collagen type I and fibronectin in fibroblasts through an intricate signaling cascade. Furthermore, at the cellular level, CNTs were shown to induce plasma membrane remodeling via rearrangement of the actin cytoskeleton and clathrin [38,41]. In addition to cytokines, matrix metalloproteinases (MMPs) play an important role in tissue remodeling; however, MMP regulation by CNTs is less studied yet. A recent study by Meng *et al.* [44] showed that naїve macrophages secreted MMP-9 and VEGF in the presence of CNTs. MMP-9 is a known key factor in TGFβ activation and extracellular matrix remodeling in normal physiology but also in the pathophysiology of lung disease [44]. In addition to MMP-9 production, it has been shown that long-term, *i.e.*, 4 months, exposure of mesothelial cells to both SWCNTs and MWCNTs increased MMP-2 expression and activity, which was involved in cell migration and invasion [45]. Poulsen *et al.* [46] showed that MWCNTs of different sizes (short: 67 ± 26.2 nm and long: 4.05 ± 2.4 μm) at either 3 days or 28 days post-exposure, induced lung remodeling regardless of the dose (54 or 163 μg/mouse). In addition to fibrosis genes of the MMP family (*Mmp-12*, *Mmp-13*, *Mmp-14*), MWCNT also upregulated the expression of tissue inhibitors of metalloproteinase (*Timp-1*, *Timp-2*, *Timp-3*, *Timp-4*), and TFGβ receptors signaling (*Tgfbr-1*, *Tgfbr-3*). Long MWCNTs were more potent in inducing gene expression than short MWCNTs. MWCNTs have also been shown to induce airway tissue remodeling via cyclooxygenase-2 (COX-2). Sayers *et al.* [47] showed that MWCNT potentiated allergen-induced increase in Th2, Th1, Th17 cytokines, prostaglandin D2, thromboxane B2, inflammation, and mucus-cell metaplasia in the lungs of COX-2(-/-) mice compared to wild type mice. However, no differences in airway fibrosis were noted between COX-2(-/-) and wild type mice. Similarly, Mizutani *et al.* [48] reported that MWCNTs sensitized airways in OVA-challenged mice via airway resistance, inflammation, and goblet cell hyperplasia, and induced a biphasic increase in airway responses and antigen-specific antibodies. Biphasic acute airways’ responses to CNTs manifested in the form of inflammation and inflammatory cells accumulation and chronic responses like lung fibrosis were also reported by Mercer *et al.* [37,49]. Furthermore, Wang *et al.* [50] showed a role of IL-33 in MWNCT-induced airway constriction, inflammation, and fibrosis. Il-33 signaling mediates allergen-induced Th2 cytokines which induce asthma. Asthma, a chronic airway inflammation and hyper-responsiveness is a major public health concern. Approximately 10% of the U.S. population and approximately 300 million people worldwide are affected by asthma [51].

These studies indicate that CNTs induce airways resistance in animal models and this should be taken into consideration as CNTs are being rapidly incorporated into numerous nanoproducts. Thus, it is prudent to strongly suggest the adoption of strict prevention strategies and workplace exposure controls in workplaces dedicated to CNTs/CNFs manufacture.

### 4.3. Genotoxicity

The potential of CNTs and CNFs to induce genetic alterations has been investigated in different system models; most often the results were partially dependent on the type and physical characteristics of the nanomaterial tested [52,53,54]. Although SWCNTs have been shown to induce genetic alterations, no tumors have been reported in any study [55,56]. However, some MWCNTs have been shown to induce genetic damage, but only MWCNT Mitsui 7 has been shown to induce carcinogenicity via induction of mesothelioma (reviewed by Toyokuni, [54]). A study by Sargent and coworkers [57] showed that MWCNTs induced genotoxicity; however in a subsequent study in [58], the same MWCNTs did not induce tumors unless there was an initiator. The latter study is crucial to extrapolate to human exposure, since many humans are exposed to mutagens including cigarette smoke, chemicals such as benz[a]anthracene, radon, naphthalene, diesel exhaust benzo[a]pyrene, and others. If these materials can serve as initiators, humans exposed to MWCNTs could develop tumors [58,59,60].

The potential of CNFs to cause DNA damage was initially evaluated in Chinese hamster lung fibroblast cells, V79, using the Comet assay and micronuclei formation (MN) procedures by Kisin *et al.* [61]. In this study, CNFs induced DNA damage but no differences were found between CNF or asbestos at similar doses (3 or 48 μg/cm^2^) and exposure times (3 or 24 h). CNF and asbestos dose-dependently induced MN formation in V79 cells. In the same study, CNF aneugenic or clastogenic effects were evaluated in human small airway epithelial cells (SAECs) in comparison with asbestos. CNF increased both aneugenic and clastogenic events and could be found inside the nucleus of SAECs. The mechanism by which CNFs induce genotoxicity is not known yet, however, direct physical interactions of nanofibers with mitotic machinery or with chromosomes during cell division leading to aneuploidy are possible. Aneuploidy is the result of imbalanced chromosome separation during cell division leading to the formation of daughter cells with an abnormal chromosome number. DNA damage and adduct formation are related to the generation of ROS via the Fenton reaction. The presence of transition metals such as Fe in CNFs, has been found to generate ROS; therefore, the genotoxic effects of CNF may be partially accounted for by ROS generation [61]. A major health concern of CNFs is further supported by findings in the study by Kisin *et al.* [61] where CNF genotoxicity was comparable to asbestos and CNFs were more potent than SWCNTs. In addition to ROS-mediated DNA damage, CNTs physically interact with DNA, particularly MWCNTs which have been shown to exhibit preference toward G-C rich DNA regions [24,62,63,64,65,66,67]. CNTs have been shown to induce DNA strand breaks, which can either be caused by the direct effects of ROS or apoptosis, and by the activity of endonucleases that are activated by the formation of DNA adducts [52,66]. Both SWCNTs and MWCNTs induce DNA strand breaks, PPAR activation, and formation of γH2AX foci in lung epithelial cells [30,31,52,57,58,66,67,68,69,70]. Sargent and co-authors [57,58] observed the induction of aneuploidy, formation of three spindle poles, microtubules and centrosome fragmentation, and accumulation of cells in the G2/M phase in SAEC exposed to a dose that would be a worker-relevant exposure dose of SWCNTs (0.024 μg/cm^2^). Similarly, Siegrest and co-authors [67] obtained similar results when MWCNTs were tested on the same cell type, SAECs, with the exception of the accumulation of cells in G1/S phase rather than in the G2/M phase as was the case of SWCNTs. Mechanistically, these studies indicate physical association of SWCNTs and MWCNTs with DNA, mitotic spindles, and centrosomes thus impeding proper chromosome segregation.

Long-term post-exposure of mice (C57BL/6) to SWCNTs via inhalation or aspiration, and CNFs via aspiration demonstrated that SWCNTs induced K-ras mutations regardless of the mode of administration, whereas CNFs induced such mutations only at very high doses [56]. However, despite oncogenic K-ras mutations, no tumors were found after one year in mice exposed to SWCNTs, CNFs, or asbestos, regardless of the mode of administration. Furthermore, *in vivo* studies with MWCNT-induced mesothelioma showed effects on gene stability (Cdkna2a/2b) and protein expression (vimentin and podoplanin) [58,71,72].

These findings suggest that in addition to particle chemistry, other characteristics such as physical properties, and the physiological interaction of CNTs/CNFs via administration (inhalation or aspiration) also modulate pathological responses [56]. However, these studies clearly indicate that although CNTs/CNFs induce genotoxic insults, no tumors were initiated in the animals unless there was an initiator, thus these findings should be taken with prudence.

### 4.4. MicroRNA Regulation

The role of microRNA in mediating molecular carbon-based nanoparticles toxicity is relatively unknown. microRNAs are short noncoding RNA strands that regulate post-transcriptional gene expression in plants, animals, and humans [73]. The relationship between MWCNTs and microRNA was first studied by Zhao *et al.* [74]. In this study, the authors exposed *Caenorhabditis elegans* (*CE*) to different concentrations of MWCNTs (0.1, 1, and 10 mg/L) and identified microRNA targets for MWCNTs using next-generation sequencing analysis. MWCNTs differentially dysregulated 55 microRNAs, of which 21 were up-regulated and 34 down-regulated. Using a prediction approach, the MWCNT-dysregulated microRNAs were mainly targeting genes involved in biological processes associated with development, cell adhesion, cell cycle, and immune response. The functional relationship between dysregulated microRNAs and MWCNT toxicity was also studied using a gain-of-function approach. Gain-of-function of microRNA-51 resulted in resistance to MWCNT toxicity whereas loss-of-function of microRNA-259 resulted in susceptibility of MWCNT toxicity. Although no specific mRNA targets for these dysregulated microRNAs were identified in the study, the data suggests that human exposure to MWCNTs could have complex effects on gene expression. Similarly, Nymark *et al.* [75] showed that exposure of BEAS-2B cells to MWCNTs lead to mitochondrial membrane potential (MtMP) dysfunction, regulation of over 300 genes of which 26 genes were linked to mitochondrial function, and a small microRNA signature. Of the four microRNAs signatures, miR-1275 negatively correlated with MtMP-dysfunction genes. These studies show that microRNA are involved in regulating CNT toxicity and *in vivo* translocation, and may be useful in circumventing CNTs toxicity for potential biological uses.

## 5. CNT-Induced Toxicological and Pathological Responses

### 5.1. Airway Hypersensitivity

Ultrafine particles including CNTs have been shown to induce allergic responses in several experimental model systems [76,77]. Intratracheal instillation of MWCNTs caused an allergic response in mice with increased populations of B-cells in the spleen and blood, and increased levels of Th2-cytokines (IL-4, Il-5, IL-10) and IgE [78]. MWCNT also aggravated allergic airway inflammation via infiltration of inflammatory cells and antigen presenting cells in the bronchial epithelium; increased Th cytokines/chemokines; and increased IgG1 and IgE levels [79]. Another study by Rydman *et al.* [80] found that in short term inhalation of two types of MWCNTs (rod-like rigid and flexible tangled) only the rigid type induced allergic-like-airway responses marked by increased eosinophil infiltration, mucus hypersecretion, and Th2 cytokines expression. Intratracheal administration of SWCNTs with an allergen to mice exacerbated Th2 cytokine levels in the lungs and increased IgG1 and IgE in the serum [81,82]. In a mouse model of asthma, MWCNTs induced an influx of inflammatory cells, increased mucus and IgG1 production, and increased the activation of inflammatory genes IL-13, Il-25, IL-33 and GM-CSF [43].

Investigations comparing the effects of CNFs and CNTs on airway hypersensitivity showed that CNFs increased adjuvant-mediated IgE production, whereas CNTs (SWCNTs and MWCNTs) increased IgE production and eosinophil recruitment to the lungs [83]. In this study, the authors showed that some of the CNFs had high Ni levels, but induced a medium allergic response as opposed to MWCNTs which were low in Ni, but powerful in inducing allergic responses. A more recent study by Thompson *et al.* [40] showed a differential activation of white blood cells (WBCs) in a murine-model of allergen-induced airways remodeling. Ovalbumin (OVA) alone increased the number of eosinophils while the addition of MWCNTs increased only neutrophil numbers in bronchoalveolar lavage. Mechanistically, these authors showed a role of STAT1 in MWCNT-induced airway remodeling via IL-13. MWCNTs increased goblet cells hyperplasia and airway fibrosis in mice bearing a deletion of STAT1 and OVA-sensitized mice. Molecularly, MWCNTs increased IL-13, TGFβ, and TNFα; and decreased IL-10 [40]. These studies clearly show the potency of CNTs over CNFs in inducing allergic responses, mostly due to their structure rather than due to metallic contaminants [83].

### 5.2. Inflammatory Responses

Acute and chronic inflammatory responses to CNTs have been documented in numerous *in vitro* and *in vivo* studies [35,40,84,85,86,87,88]. *In vivo* inflammatory responses were primarily characterized by the recruitment of lymphoid cells into the bronchial epithelium, lung parenchyma, and lung perivascular tissue [35,56,89,90]. Regardless of the mode of administration (e.g., inhalation, instillation, intratracheal instillation), MWCNTs induced the recruitment of eosinophils, neutrophils, macrophages, and antigen-activated dendritic or goblet cells [10,39]. Poulsen *et al.* [46] showed that MWCNTs induced the activation of inflammatory genes TNFα, CCL and CXCL chemokines, and increased the levels of serine protease inhibitors (SERPINs), serum amyloid A3, TNFα, and Il-1β regardless of their physical size (short or long).

### 5.3. Fibrogenic Responses

Numerous studies have demonstrated the fibrogenic potential of CNTs either in *in vitro* or *in vivo* models (Figure 1) [37,46,84,91,92]. Although particle settlement in the lungs is partly determined by physical properties, *i.e.*, diameter and length, studies have shown that SWCNTs possess a greater fibrotic potential than MWCNTs [37,92]. However, Mercer *et al.* [37] showed that MWCNTs with a mean size of 3.9 μm × 49 nm deposited at approximately 8% in the alveolar septa at 56 days post-exposure and progressively increased collagen deposition in the lungs over a period of 11 months [37,49]. Long term post-exposure studies of CNTs showed the presence of MWCNTs in the lung interstitium; however, most of the MWCNTs were of small size and seemed to be less potent in inducing lung fibrosis than SWCNTs [37,39,56].

### 5.4. Tumorigenic Responses

Whether CNTs stimulate the growth of lung tumors still remains unknown; however, several studies suggest that CNTs may act as tumor promoter. Shvedova *et al.* [10,35] showed that one single acute exposure to SWCNTs resulted in lung tumor burden but was conditional on SWCNTs interactions with myeloid-derived suppressor cells (MDSCs) and TGFβ production. Studies investigating the tumorigenic effects of MWCNTs showed no lung cancer but mesothelioma, which at times was dependent on the type of MWCNTs involved (Figure 1). Muller *et al.* [88] reported that short MWCNTs (<1 μM) administered intraperitoneally did not induce mesothelioma. Similarly, Nagai *et al.* [88,93] reported no mesothelioma following thick or tangled MWCNTs (diameter: 150 and 15 nm) administration, but mesothelioma with thin MWCNTs (diameter 50 nm) intraperitoneally administered to rats. Takagi *et al.* [94] showed the induction of mesothelioma in p53-deficient mice. Sargent *et al.* [58] showed that inhalation of MWCNTs (Mitsui-7) induced lung neoplasms in mice only if mice were pretreated with the neoplasm initiator methylcholanthrene (MCA). However, mice exposed to MCA followed by MWCNTs inhalation developed malignant sarcomatoid mesotheliomas morphologically appearing as nodular masses composed of polygonal to spindloid cells in the diaphragm [58].

To further investigate the tumorigenic mechanism of CNTs, Luanpitpong *et al.* [95,96] showed that chronic exposure of human lung cells to SWCNTs for 6 months induced the appearance of cancer stem cells-like cells, which when implanted in mice promoted tumor development. The mechanism by which SWCNTs promoted the formation of cancer stem cells-like cells was partly via p53 inactivation and caveolin-1 over-expression [97].

## 6. Human Exposure to CNTs/CNFs

Few studies have reported human exposure to CNTs/CNFs. In 2012, Dahm *et al.* [97] measured exposure levels to CNTs/CNFs at six primary and secondary manufacturing sites. Using personal breathing area and area sampling the authors found most of the collected samples were below the REL (7 μg/m^3^) and only two tested sites had higher exposure limits than REL. The 7 μg/m^3^ REL was the initial exposure level set by NIOSH in 2010 [98].

The actual effect of MWCNTs on human health was first presented by Lee *et al.* [99] for a U.S. MWCNTs manufacturing site. The authors surveyed workers’ health in a workplace where MWCNTs are manufactured. A higher level of elemental carbon was found in the personal worker area than in the sampling area (6.2–9.3 μg/m^3^ and 5.5–7.3 μg/m^3^, respectively). The exhaled breath condensate of manufacturing workers contained higher levels of oxidative stress markers (MDA, 4-HHE, and *n*-hexanal) and higher blood molybdenum than office workers. The levels of CNTs/CNFs in the personal breathing area of workers were found to be above the proposed recommended exposure limits in secondary manufacturing sites that use the nanoparticles for commercial applications [99]. Kuijpers *et al.* [100] assessed occupational exposure of workers to MWCNTs during production and handling at a commercial facility in The Netherlands. The authors reported that in the production and handling areas, the exposure levels to MWCNTs were similar at 41 and 43 μg/m^3^ respectively, whereas in the research and development area (R&D) and the office area, exposure levels of MWCNTs were at 5 and 7 μg/m^3^, respectively. However, in 2013 The U.S. agency NIOSH has recommended an occupational exposure limit (REL) of 1 μg/m^3^ CNT which initially was set at 7 μg/m^3^ [101] for an 8 h time-weighted average of elemental carbon EC, the respirable fraction of CNT. Although, in the above studies, the exposure level of MWCNTs was much higher than the REL, neither study indicates whether the workers were wearing personal protective equipment. Han *et al.* [102] showed that use of engineering controls effectively decreases airbone MWCNTs during synthesis and processing in a laboratory setting. Methner [103] showed that local exhaust ventilation decreased MWNCTs during reactor cleaning by 88%, and Regasamy *et al.* [104] showed that face respirators with 95 or 100 series filters effectively captured 95% of nanoparticles 4–30 nm in size.

## 7. Conclusions and Perspectives on CNTs

In summary, the experimental data presented in this review clearly shows that CNTs/CNFs induce respiratory pathologies in animal models, although some studies (a few) report negative results (Figure 1 and Table 1), and that CNTs/CNFs are released during production and handling. Moreover numerous studies conducted over the past two decades since the discovery of CNTs in 1991 have provided a wealth of scientific knowledge on CNT/CNF interactions with biological systems and have contributed to development of methods of handling, limits of exposure, and toxicity outcomes, and importantly classification of one type of MWCNTs as carcinogen. Among the different types of MWCNTs presented in this article, based on the experimental data, MWCNT Mitsui 7 is classified as a carcinogen [105,106].

With regards to the carcinogenicity of CNTs/CNFs, there are no studies which show that chronic exposure to CNTs/CNFs leads to the development of lung cancer. Two studies, Shvedova *et al.* [56] and Sargent *et al.* [58] show the absence of lung tumors induced only by CNTs/CNFs (Table 1). However, the study by Sargent *et al.* [58] clearly shows that if a tumor is initiated, MWCNTs promote lung adenocarcinoma. This study is most likely to reflect humans, since people smoke or have genetic predisposition, *etc.*, thus in such situations, CNTs may accelerate tumor progression. This is also supported by numerous *in vitro* studies which point out that CNTs activate molecular mechanisms associated with tumorigenesis and since typical fiber-induced lung cancer in humans is 30–40 years, it is prudent to advise the adoption of strategies and implementation of exposure controls in environments where CNTs/CNFs are present. Given that the production and applications of CNT-based nanoproducts will likely increase and thus the presence of CNTs in the environment either from product wear, disposal, or manufacturing will likely increase. Therefore, CNTs might become more bioavailable andthis could enhance the potential for adverse respiratory effects. Since many chronic lung diseases, *i.e.*, fibrosis and chronic obstructive pulmonary disease (COPD), are time-dependent, novel and innovative approaches to circumvent the advent of CNT accumulation in the environment need to be considered. Just as the studies by Martino *et al.* [110] and Favero-Longo *et al.* [111] showed that soil fungi and lichens were detoxifiers of asbestos via chelation or removal of reactive metal iron, the scientific community must envision the development of novel protocols leading to nanobioremediation. For example, Yang *et al.* [112] developed a chemical approach based on the use of Ca^2+^ and paper filtration to extract CNTs from an aqueous environment, while Zhang *et al.* [113] showed that pristine or oxidized (O^−^) MWCNTs at low doses enhanced bacterial growth and biodegradation of organic molecules in an aqueous environment; however, high doses inhibited bacterial growth. In addition to nanobioremediation, the use of CNTs to increase microalgae biomass for sustainable energy production has also been proposed [114]. Based on the history of asbestos and its human health effects, the scientific community may explore the use of procedures developed for other environmental contaminants, such as asbestos, as a guide to more safely produce and process nanomaterials [115]. Although asbestos has been banned in the USA and other countries, it still remains an environmental hazard. Consequently, research is still being conducted to develop methods to reduce asbestos contamination. We can potentially use this knowledge to design and post-process CNTs as a preemptive approach to minimizing nanodiseases and to avoid future asbestos-like phenomena.

## Figures and Tables

**Table 1:** List of some of the *in vivo* studies which show CNTs/CNFs toxicity on the respiratory system.

Year	Types of CNTs/Sources	CNTs Length	Assay Type	Mode of Administration	Dose	Exposure Time	Outcome	Reference
***MWCNTs negative results***								
2009	MWCNT +/− defects	<1 μm	Wistar rats	I.P.	2, 20 mg/animal	24 months	No mesothelioma	[88]
2010	MWCNT (MWNT-7, Lot # T050831-01, Mitsui & Co. Ltd. 9, Tokyo, Japan)	1.5 μm	SD rats	I.T.	0.04, 0.2, 1 mg/kg	3, 7 days; 1, 3, 6 months	Negative interstitial tissue, absence of fibrosis, MWCNT + Macrophages in alveoli. Lower case for macrophages.	[107]
***MWCNTs positive results***								
2004	CNT (Raw and Purified HiPco NTs, Rice University, Huston, TX, USA); CNT (CaboLex Inc., Lexington, KY, USA)	<1 μm	mice	I.T.	0.1, 0.5 mg/animal	7, 90 days	Epitheloid granulomas, interstitial inflammation, lung necrosis; high dose induced 60% death	[89]
2009	MWCNT (Bussan Nanotech Research, Ibaraki, Japan); SES Research (TX, USA)	3–30 μm, several μm	ICR mice	I.T.	25, 50 μg	6 weeks	Exacerbation of allergic murine airway inflammation	[79]
2012	MWCNT (Mitsui MWCNT-7, No. 060125-01k, Tokyo, Japan)	<5 μm	p53 +/− mice	I.P.	0.3 mg/animal	1 year	Mesothelioma	[94]
2013	MWCNT (MWCNT-7, lot # 06122031, Hodogaya, Japan)		C57BL6J	Inhal.	5 mg/m^3^, 5 h/d, 4 d/wk, 12 d	1, 14, 84, 168, 336 days	Pulmonary inflammation, progressive collagen in alveolar regions after 336 days	[49]
2013	CNF (Statoil and Elkem Carbon AS, Kristiansand, Norway) SWCNT (Sigma Aldrich, St. Louis, MO, USA, Cat. # 636797); MWCNT (Sigma-Aldrich, St. Louis, MO, USA, Cat. # 636487)	5–10 0.5–100 0.5–200 μm	BALB/cAnNCrl Mice	Intra-nasal	400 μg/mouse	26 days	Modulation of airway responses to allergens; CNT more potent than CNF	[83]
2014	MWCNT (Mitsui-7, MWNT-7, lot #061220-31, Hodogaya, Japan) MCA, MWCNT, MCA + MWCNT	0.5–5 μm	B6C3F1	Inhal.	5 mg/m^3^, 5 h/d, 5 d/wk, 15 d	17 months	MCA potentiated MWCNT adenocarcinoma MWCNT alone did not induce adenocarcinoma	[58]
2015	MWCNT	5.53–6.19 μm	F344 rats	Inhal.	0.2, 1, 5 mg/m^3^	13 weeks	granulomatousa in females at 1 and 5 mg/m^3^; in males at 0.2 mg/m^3^ fibrosis	[108]
2015	MWCNT CNT_small_ (NC700, Nanocyl, Sambreville, Belgium; 13% impurities); CNT_large_ (NM-401, European Joint Research Centre, Ispra, Italy; 3% impurities)	0.85, 4.05 μm	C57BL/6 mice, female	I.T.	18, 54, 162 μg/animal	1, 3, 28 days	Similar inflammatory and acute responses to both types; stronger fibrotic response to CNT_large_ than CNT_small_	[46]
***SWCNTs positive results***								
2010	SWCNT: CNI, (USA); SES Res. (USA)	1–15 μm	ICR mice	T.	50 μg	6 weeks	Exacerbation of allergic murine airway inflammation	[82]
2014	SWCNT (HiPco, Unidym, Sunnyvale, CA, USA); CNF (Pyrograf Prod., USA) Asbestos (UICC, USA)	1–3 μm, 5–30 μm, 2–30 μm	C57BL/6 mice	Pharyngeal aspiration, inhalation	40 μg/animal 40, 120 μg/animal 120 μg/animal 5 mg/m^3^, 5 h/4 d	1 year	All particles induced chronic bronchopneumonia, pulmonary fibrosis. CNF > asbestos > SWCNT inflammation, SWCNT were the most fibrogenic, CNF, SWCNT induced K-ras mutations, No tumors	[56]
2015	SWCNTs (Tech. Res. Assoc. for SWCNT, Japan)	CNT-1 (0.51 μm, short); CNT-2 (1.67 μm, long/thick)	Wistar rats	I.T.	0.18, 1.8 mg/kg	1, 3, 7, 30, 90 days	Lung focal inflammation, neutrophil in alveoli, lung macrophages and cell derby in alveoli, higher CNT burden in CNT-1 than CNT-2 at 90 days	[109]

CNTs, carbon nanotubes; CNFs, carbon nanofiber; IT, intratracheal instillation; Inhal, inhalation; IP, intraperitoneal; MCA, methylcholanthrene.
